# The critical role of iron homeostasis in neurodegenerative diseases

**DOI:** 10.4103/NRR.NRR-D-24-01382

**Published:** 2025-04-29

**Authors:** Tiantian Liang, Jiasen Xu, Yan Zhu, He Zhao, Xiaoyu Zhai, Qi Wang, Xiaohui Ma, Limei Cui, Yan Sun

**Affiliations:** 1 Department of Otorhinolaryngology, Head and Neck Surgery, Yantai Yuhuangding Hospital, Qingdao University, Yantai, Shandong, China; 2 Shandong Provincial Key Laboratory of Neuroimmune Interaction and Regulation, Yantai, Shandong, China; 3 Shandong Provincial Clinical Research Center for Otorhinolaryngologic Diseases, Yantai, Shandong, China; 4 Yantai Key Laboratory of Otorhinolaryngologic Diseases, Yantai, Shandong, China

**Keywords:** ferroprotein, neurodegenerative diseases, iron homeostasis, iron, iron regulatory proteins

## Abstract

Neurodegenerative diseases are prevalent conditions that greatly impact human health. These diseases are primarily characterized by the progressive loss and eventual death of neuronal function, although the precise mechanisms underlying these processes remain incompletely understood. Iron is an essential trace element in the human body, playing a crucial role in various biological processes. The maintenance of iron homeostasis relies on the body’s intricate and nuanced regulatory mechanisms. In recent years, considerable attention has been directed toward the relationship between dysregulated iron homeostasis and neurodegenerative diseases. The regulation of iron homeostasis within cells is crucial for maintaining proper nervous system function. Research has already revealed that disruptions in iron homeostasis may lead to ferroptosis and oxidative stress, which, in turn, can impact neuronal health and contribute to the development of neurodegenerative diseases. This article primarily explores the intimate relationship between iron homeostasis and neurodegenerative diseases, aiming to provide novel insights and strategies for treating these debilitating conditions.

## Introduction

Neurodegenerative diseases are a group of age-related conditions characterized by the progressive loss of structure or function in neurons and glial cells. These diseases typically manifest as the selective dysfunction and persistent loss of specific neuronal populations, leading to impairments in the nervous system and ultimately resulting in impaired memory, cognition, behavior, sensation, and/or motor function (Collingwood and Davidson, 2014; Liu et al., 2018; Menéndez-González, 2023). The onset of neurodegenerative diseases is associated with a variety of factors, including genetic, environmental, and biochemical changes, and has a particularly close relationship with pathological alterations in proteins (Kovacs, 2016; Sousa et al., 2020; Tian et al., 2024).

Neurodegenerative diseases are a complex group of conditions that can be classified based on their association with protein abnormalities, clinical manifestations, and underlying mechanisms. First, many of these diseases are intrinsically linked to the abnormal aggregation or processing of specific proteins. Alzheimer’s disease (AD), for instance, is associated with the abnormal accumulation of amyloid-β (Aβ) and tau proteins, which disrupt normal brain function. Similarly, Parkinson’s disease (PD) is characterized by the aggregation of alpha-synuclein (α-syn), which leads to the degeneration of dopaminergic neurons (Kovacs, 2017; Long et al., 2023). Clinically, neurodegenerative diseases can be divided into movement and cognitive disorders. Movement disorders, such as PD and Huntington’s disease (HD), primarily affect motor functions and are often accompanied by symptoms such as tremors and rigidity. These conditions can greatly impair a person’s physical and movement abilities. Cognitive disorders, typified by AD, are marked by a decline in memory and cognitive functions that lead to difficulties in thinking, reasoning, and memory retention. From an etiological standpoint, neurodegenerative diseases are categorized as hereditary and non-hereditary. Hereditary diseases, including HD and familial AD, are caused by specific genetic mutations that result in progressive neuronal damage. These mutations can be passed down through generations, predetermining an individual’s risk for developing the diseases. On the other hand, non-hereditary diseases, such as senile dementia and multiple sclerosis (MS), have more complex pathogenesis, with environmental factors and lifestyle choices potentially playing crucial roles (Kovacs, 2016; Menéndez-González, 2023). These diseases can arise from a combination of genetic predisposition and external influences, making them more challenging to predict and treat.

**Figure 1:**
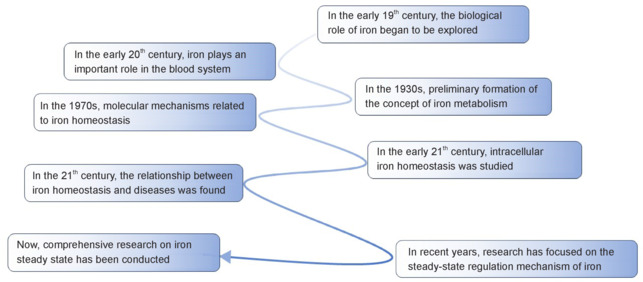
Chronological events in the study of the iron steady state.

In the early 19^th^ century, scientists linked iron deficiency to diseases such as anemia. Early in the 20^th^ century, the role of iron in the function of hemoglobin was confirmed. In the 1930s, basic iron metabolism concepts began to emerge, and by the mid-to-late 20^th^ century, biological iron absorption mechanisms were clarified. In the 21^st^ century, scientists gained a greater understanding of iron homeostasis regulation mechanisms, and their role in diseases and treatments is the subject of ongoing research (Donovan et al., 2006; Sheftel et al., 2012; Coates and Cazzola, 2019; Camaschella et al., 2020; Koleini et al., 2021; **Figure 1**).

Iron is one of the most abundant transition metals in the human body, occurring in two oxidation states: Fe^2+^ and Fe^3+^. Not only is iron a key component of important molecules such as hemoglobin, myoglobin, and cytochrome but it is also involved in a variety of biological processes (Li et al., 2010). Iron plays an important role in the function of enzymes such as catalase and peroxidase, especially in oxygen metabolism, the mitochondrial respiratory chain, and the electron transport process (Silvestri et al., 2023). Iron in the body is primarily absorbed from the diet, with certain foods—such as those rich in vitamin C—facilitating its uptake. In contrast, other substances, including calcium and certain phytates, may impede iron absorption. Once absorbed, iron is transported in the bloodstream via transferrin; it is reduced from Fe^3+^ to the more absorbable Fe^2+^ by reductases during transport, then enters the cytoplasm via the divalent metal transporter (DMT1) (Baringer et al., 2023). There is no specific excretion mechanism for iron in the body, and its loss usually occurs through bleeding or cell shedding (Pantopoulos, 2004). Although iron is an essential trace element, in excess, it can lead to oxidative stress and cellular damage. Therefore, iron levels in the body must be tightly regulated through strict mechanisms to prevent the toxic effects of its accumulation (Bogdan et al., 2016).

Iron plays a variety of roles in maintaining neural function and is essential for the immune system, DNA synthesis, and gene regulation. Despite its low bioavailability, iron is indispensable for the normal physiological functioning of organisms. Iron has highly active redox properties, allowing it to readily convert between oxidation states and participate in electron acceptance and donation. However, it is these properties that make excess iron potentially toxic *in vivo* (Lane et al., 2018).

Cells have developed complex regulatory mechanisms to maintain a balance among iron absorption, storage, and excretion and to ensure the maintenance of intracellular iron homeostasis. Iron is an essential component for neuronal generation and differentiation and plays a crucial role in neural development, particularly during early life stages. Research shows that changes in iron concentration in the brain are closely related to the development and function of neurons (McCarthy et al., 2022; Georgieff, 2023). Early in life, iron deficiency is associated with impaired cognitive function, which may lead to decreased attention, learning, and memory abilities. It may also have long-term effects on behavior and neural development, and could even be irreversible (Beard, 2003). The balance of iron in the brain is crucial for normal cognitive development and function. Excessive or insufficient iron can lead to the dysfunction of the nervous system, while appropriate iron levels can enhance the antioxidant capacity of nerve cells and protect them from oxidative stress damage (Carlson et al., 2009; Wang et al., 2019b; McCann et al., 2020).

Understanding the relationship between iron homeostasis and neurodegenerative diseases is of great significance for revealing the morbidity mechanisms of neurodegenerative diseases and the search for new therapeutic targets. This review mainly focuses on the relationships between iron homeostasis and neurodegenerative diseases such as AD, PD, Friedreich’s ataxia (FRDA), HD, amyotrophic lateral sclerosis (ALS), MS, and neuroferrotinopathy (NF). Further studies on the role of iron homeostasis in neurodegenerative diseases are expected to provide new ideas and methods for the treatment and prevention of these conditions.

## Retrieval Strategy

For this narrative review, we searched the PubMed database for studies published between 1988 and 2024 on the relationship between iron homeostasis and neurodegenerative diseases, mainly using the keywords “iron homeostasis,” “neurodegenerative diseases,” and “iron” in combination with “Alzheimer’s disease,” “Parkinson’s disease,” “Huntington’s disease,” “Friedreich’s ataxia,” “amyotrophic lateral sclerosis,” “multiple sclerosis,” and “neuroferritinopathy,” respectively. The results of the search were initially screened by title and abstract to ensure that the selected literature closely aligned with iron homeostasis and neurodegenerative disease research. Finally, after reading and analyzing the full text, 147 articles were included as references in the review, most of which were published in the past 5 years (2019–2024) to ensure the timeliness and relevance of the review content.

## Iron Homeostasis and Neurodegenerative Diseases

### Iron homeostasis

**Figure 2:**
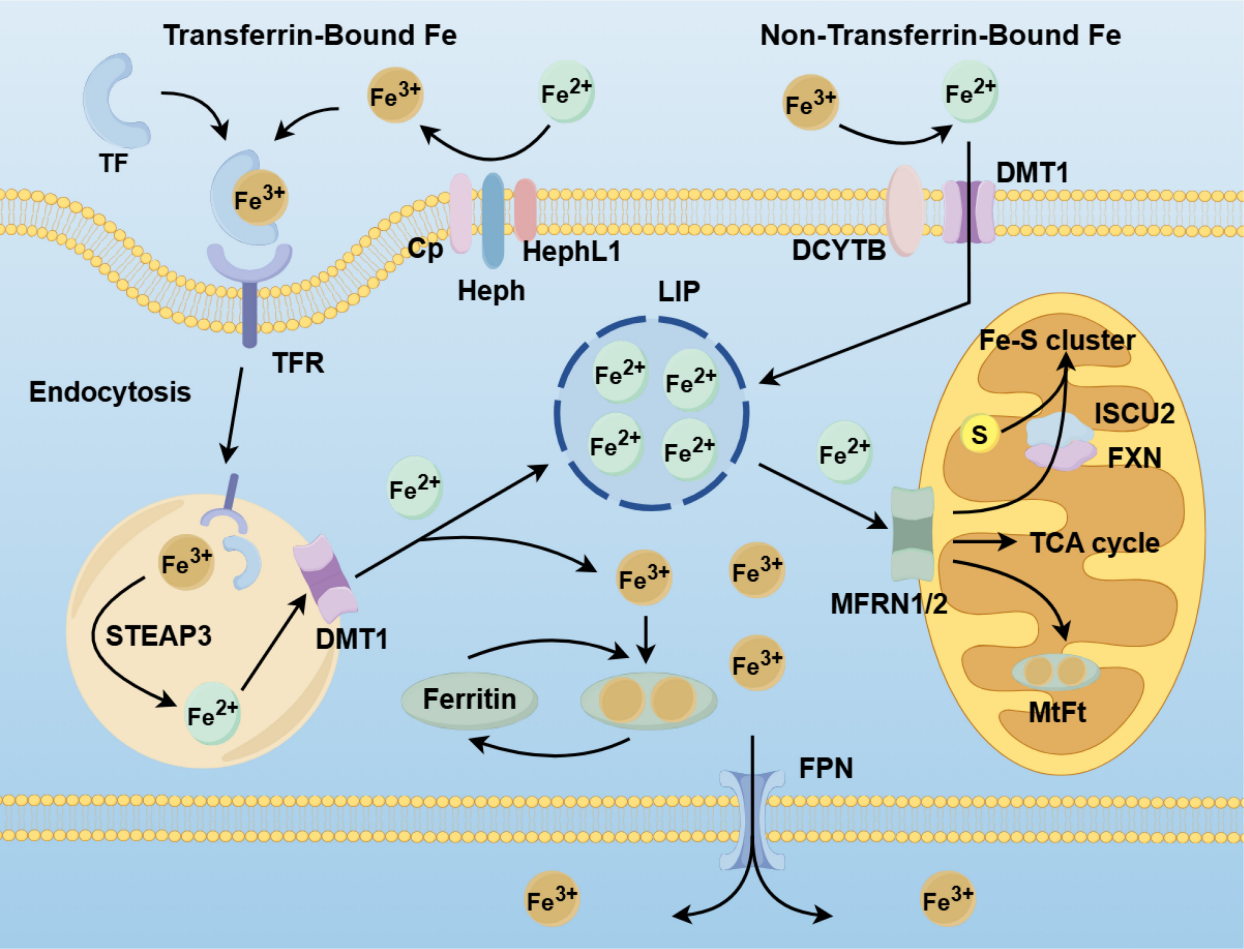
Non-heme iron is classified as transferrin-bound iron or non-transferrin-bound iron (NTBI). Divalent metal transporter (DMT1) promotes the cellular uptake of ferrous iron, which is synonymous with NTBI. Iron is reduced to the ferrous form in response to duodenal cytochrome B (DCYTB). Iron binds to transferrin, which then interacts with transferrin receptors, and eventually, iron-transferrin complex endocytosis is internalized into the cell. Within the confines of endocytic vesicles, iron is released by the six transmembrane epithelial antigen (STEAP3) of prostate 3 and is reduced back to its ferrous state. DMT1 subsequently mediates the export of ferrous iron from vesicles to the cytoplasm. In the cytoplasm, ferrous iron constitutes the unstable iron pool (LIP), and iron passing through ferroprotein (FPN) is exported from the cell. LIP iron can be imported into mitochondria via mitochondrial ferritin (MTFN1/2) for iron‒sulfur cluster biosynthesis, the tricarboxylic acid (TCA) cycle, or storage in mitochondrial ferritin (MtFt).

Iron homeostasis refers to the dynamic equilibrium between the intake, utilization, storage, and excretion of iron within the body. The key to maintaining this balance lies in the absorption and distribution of iron, primarily regulated by the peptide hormone hepcidin, which is synthesized in the liver (Finberg, 2011; Wallace, 2016). Intracellular iron homeostasis is regulated through precise feedback mechanisms, and cells adjust their uptake and storage of iron based on their iron requirements. This regulation involves a variety of molecules and pathways that ensure a balance of iron, meeting the physiological needs of the cell while preventing excessive accumulation (Kühn, 2015; Nadimpalli et al., 2024). Under physiological conditions, almost all circulating iron is complexed with transferrin. DMT1 facilitates the cellular uptake of ferrous iron. Conversely, ferric iron undergoes reduction to its ferrous form by the action of duodenal cytochrome B, while the multicopper ferroxidases ceruloplasmin, hephaestion, and hephaestin-like protein 1 catalyze the oxidation of ferrous iron to ferric iron (Ems et al., 2025). Ferric iron then engages with transferrin, which subsequently interacts with the transferrin receptor, culminating in the endocytic internalization of the iron-transferrin complex (Kühn, 2015). Within the confines of the endocytosed vesicles, the iron is liberated and reduced back to its ferrous state by six-transmembrane epithelial antigen of the prostate 3 (STEAP3) (Ohgami et al., 2005; Feng et al., 2020). Subsequently, DMT1 mediates the export of ferrous iron from the vesicles to the cytoplasm, completing the cellular iron trafficking process. In the cytoplasm, ferrous iron constitutes the labile iron pool (LIP), which acts as an intermediary between iron input, storage, and utilization. Iron is exported from cells via the only currently known cellular iron exporter, ferroprotein (FPN) (Ems et al., 2025), and iron transport proteins play a key role in this process. Cells also use such transport proteins to regulate the influx of iron. Transferrin’s primary function is to transport iron from the intestine to the cells, while ferritin is responsible for storing excess iron for future use. The coordinated action of these two proteins allows cells to effectively manage the flow of iron under different conditions (Kühn, 2015; Anderson and Frazer, 2017). In addition to their use of transport proteins, cells, in response to feedback signals, regulate the absorption and storage of iron by modulating the stability of other mRNA related to iron metabolism (Wang and Babitt, 2019). This mechanism allows cells to adjust their metabolic activities in response to the availability of iron, finely tuning their iron balance. By doing so, cells can respond to changes in iron supply and demand, maintaining the homeostasis of iron metabolism, which is crucial for cellular health and function. Most iron in cells is transformed into ferritin (Zeidan et al., 2021; Ems et al., 2025), but LIP iron can also be imported into mitochondria for iron-sulfur cluster biosynthesis, use in the tricarboxylic acid cycle, or storage as mitochondrial ferratin (Paradkar et al., 2009; Ward and Cloonan, 2019; **Figure 2**).

Despite the physiological regulation of iron, the body excretes iron at an almost constant basal rate, which can result in its accumulation and subsequent overload (Zeidan et al., 2021). As a result, iron tends to accumulate in certain tissues as it ages (Masaldan et al., 2019). Excess iron can induce oxidative stress, leading to cellular and tissue damage (Galy et al., 2024).

### Iron regulatory proteins are key factors in iron homeostasis

In vertebrates, iron regulatory proteins (IRPs) play crucial roles in maintaining cellular homeostasis by sensing intracellular iron levels and binding to iron response element (IRE) to regulate the translation of proteins involved in iron metabolism. These cytoplasmic RNA-binding proteins exist in two forms, IRP1 and IRP2, that are widely distributed in the cortex, hippocampus, striatum, and peripheral organs (Ma et al., 2023). IRE is an mRNA stem-loop structure that serves as a target for translational control by IRP1 and IRP2 (Volz, 2021; Yao et al., 2022). Although IRP1 and IRP2 share 64% amino acid sequence homology, regulation of their RNA-binding activity proceeds through different mechanisms. IRE can be present in either the 3′-UTR or 5′-UTR of the target mRNA. Transcripts with IRE motifs in the 5′-UTR include ferritin H and L subunits, FPN, and aminolevulinic acid synthetase, whereas target mRNAs with IRE motifs in the 3′-UTR include transferrin receptor (TfR) and DMT1 (Zhou and Tan, 2017). The maintenance of iron homeostasis primarily relies on the coordinated actions of transferrin receptors and ferritin. When intracellular iron concentrations are high, the binding affinity of IRPs to IREs decreases, leading to the increased translation of ferritin, which stores excess iron. Conversely, when iron concentrations are low, IRPs bind more strongly to IREs, reducing the expression of TfR and consequently decreasing iron uptake. This bidirectional regulatory mechanism allows cells to dynamically adjust their iron storage and uptake, thereby maintaining the homeostatic balance of iron within the cell (Yu et al., 2023; Galy et al., 2024).

The dysregulation of the iron homeostasis mechanisms under the control of by IRPs is implicated in a range of diseases, including neurodegenerative disorders, cardiovascular diseases, hematological conditions, and tumors of the digestive system (Zhang et al., 2024). Within the nervous system, maintaining iron homeostasis is of paramount importance, as its imbalance is intricately linked to the onset and progression of various neurodegenerative diseases.

IRP1 is a bifunctional protein that acts as a cytosolic aconitase and an RNA-binding protein involved in the post-transcriptional regulation of iron metabolism, and the function it executes depends on intracellular iron levels: When the cellular iron level is high, IRP1 binds to the 4Fe-4S cluster, turning it into cytosolic aconitase, which no longer binds to IRE. Cytosolic aconitase then exerts its enzymatic function, i.e., reducing the regulation of iron metabolism genes (Silvestri et al., 2023). When the cellular iron level decreases, IRP1 is phosphorylated and disassociates from the Fe-4S cluster. IRP1 loses its enzymatic function and transforms into a form that can bind to IRE. By binding to the 5′-UTR of mRNA, IRP1 inhibits the translation of ferritin and reduces iron storage. At the same time, by binding to the 3′-UTR, the mRNA of TfR is stabilized, and therefore iron uptake increases (Kim et al., 1996; Zhou and Tan, 2017; Yao et al., 2022).

**Figure 3:**
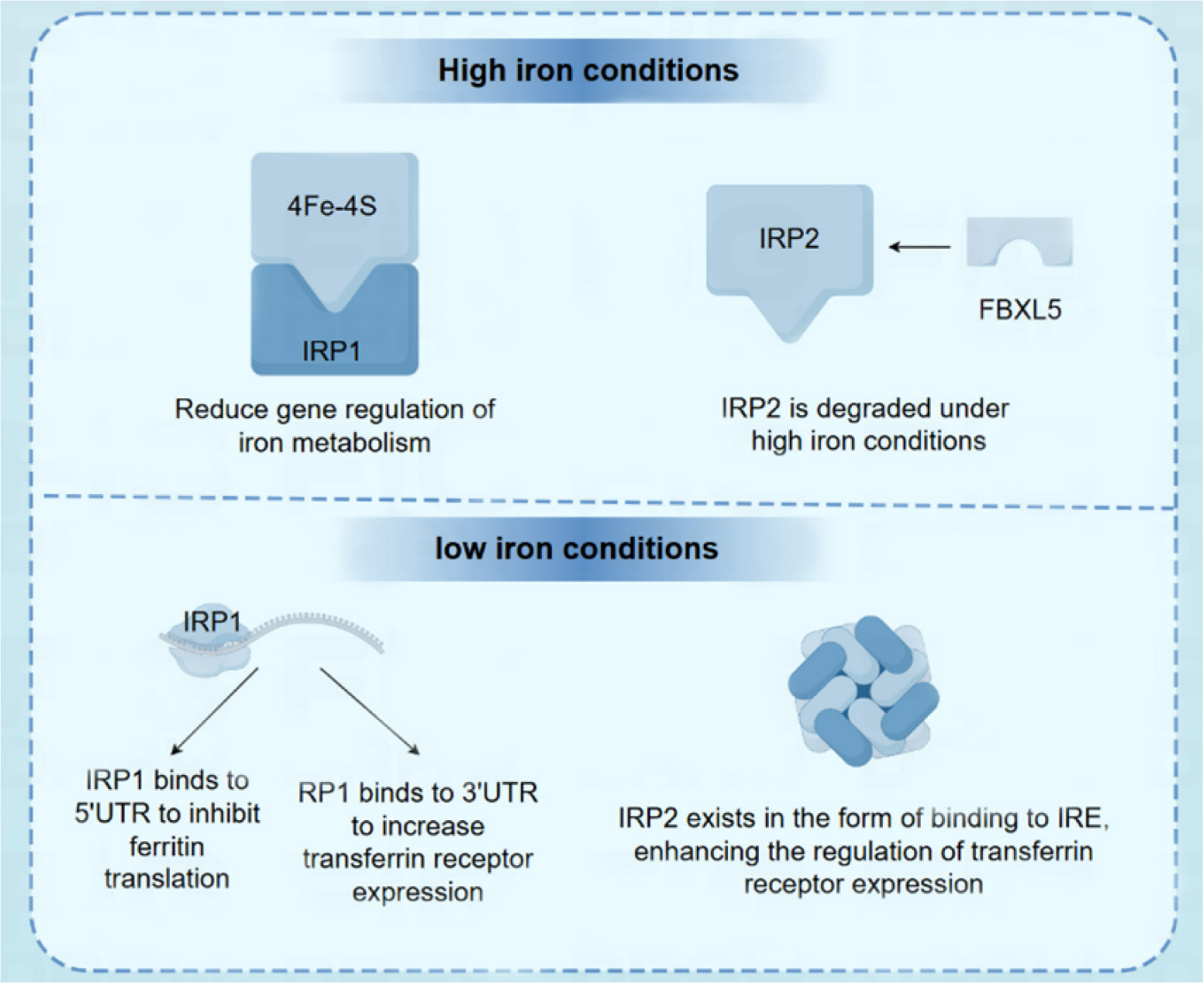
Illustration of the mechanism of action of iron regulatory protein (IRP1) and IRP2 in regulating iron homeostasis in cells. Under high-iron iron conditions, IRP1 binds to [4Fe-4S], which functions as an aconitase and reduces the regulation of iron metabolism genes. Under low-iron conditions, IRP1 loses [4Fe-4S] and transitions to an iron-responsive element (IRE)-bound form that inhibits ferritin synthesis and increases transferrin receptor 1 expression. IRP2 is mediated by F-box and leucine-rich repeat protein 5 (FBXL5) when iron is abundant and regulates gene expression by binding to IRE when iron is deficient, increasing iron uptake and storage.

One feature distinguishing IRP2 from IRP1 is the presence of a conserved 73-amino-acid extension near its N-terminus rich in cysteine and proline. Due to its structural differences, IRP2 cannot assemble iron-sulfur clusters and does not function as an aconitase (Wang and Pantopoulos, 2011). Rather, it specifically acts as an RNA-binding protein to regulate iron metabolism genes (Maio et al., 2022). IRP2 has been shown to undergo oxidation and ubiquitination prior to enzymatic degradation; it has a unique iron-dependent degradation domain that is recognized by HOIL-1, prompting it to undergo oxidation. The iron- and oxygen-dependent degradation of IRP2 is mediated by FBXL5 E3 ligase. The structural stability of FBXL5 is disrupted in iron-deficient cells, making its unable to degrade IRP2 to provide a substrate for ubiquitination, and IRP2 cannot function as a heptapeptide (Yamanaka et al., 2003; Wang et al., 2020; Yao et al., 2022, 2024b; **Figure 3**).

The expression of IRP2 in the gut and brain is important, especially in neurons. IRP2 supports the function of mitochondria and the synthesis of neurotransmitters by regulating iron uptake and storage, ensuring the normal development and function of neurons (Bengson et al., 2023). After targeted IRP2 knockout, mice exhibited neuronal loss, axonal degeneration, and myelin abnormalities with iron accumulation (LaVaute et al., 2001; Cooperman et al., 2005; Costain et al., 2019). Several studies have suggested that a loss of IRP2 may increase the risk of neurodegenerative diseases by affecting mitochondrial function, promoting oxidative stress, and interfering with the synthesis of neurotransmitters and the maintenance of myelin (Chen et al., 2020; Shen et al., 2021; Porras et al., 2024). Therefore, the targeted regulation of IRP2 may provide a potential therapeutic approach for neurodegenerative diseases.

IRPs regulate the expression of TfR, DMT1, and FPN. Tf-TfR is considered to be a major pathway for cellular iron uptake in the brain. When cells need more iron, the cellular IRP/IRE system promotes the expression of TfR and releases stored iron (ferritin) through nuclear receptor coactivator 4 activation, and the main receptor responsible for activating this pathway is TfR1. The iron is then transported across the endosomal membrane via a process mediated by DMT1, which controls ferrous iron uptake. FPN is the only known iron transporter responsible for cellular iron export (Wang and Pantopoulos, 2011; Ward and Kaplan, 2012; Lee and Hyun, 2023). IRP1 binds to IREs in the 5’-UTR of FPN mRNA during iron deficiency, blocking ribosome binding and inhibiting translation, thereby reducing FPN expression and limiting iron export from cells (Lee and Hyun, 2023).

In addition to the iron concentration, other factors also affect the activity of IRPs. The IRP/IRE system and HIF-HRE system interact with each other through iron oxygen crosstalk to jointly affect cellular iron uptake and homeostasis (Wang et al., 2010; Hirota, 2019; Yao et al., 2022). NO, through its activity as a neurotransmitter, can affect the iron metabolism function of IRP1, but its impact on IRP2 is not yet clear (Liu et al., 2019; Yao et al., 2022). IL-1, an inflammatory factor, indirectly increases the recruitment of IRP1 and enhances its binding with IRE, but does not alter the level of IRP1 protein (Kwak et al., 1995; Piñero et al., 2000a). The redox state within cells can also affect the activity of IRPs, and other factors such as iron like metals, gut microbiota, viral infections, hormones, and vitamins also affect iron homeostasis. Although these factors are not the main regulators of iron homeostasis, they may play important roles in neurodegenerative diseases.

## Iron Homeostasis in Neurodegenerative Diseases

### Central nervous system iron homeostasis

In the central nervous system, iron plays a particularly important function. It is involved in mitochondrial energy transduction, enzyme catalysis, myelination, and synaptic plasticity, as well as in the synthesis and metabolism of neurotransmitters (Devos et al., 2014; Lane et al., 2018). Iron participates in the synthesis and release of neurotransmitters, such as dopamine, norepinephrine, and serotonin, which play key roles in the transmission of information in the nervous system. Additionally, iron is essential for processes such as DNA synthesis and repair, as well as mitochondrial energy metabolism, making it crucial for maintaining the normal physiological function of nerve cells. The brain is highly sensitive to disruptions in iron metabolism, and with advancing age, iron levels in the brain tend to increase. Furthermore, brain iron accumulation occurs in regions implicated in disease pathology and may accompany oxidative stress, inflammation, and cell death (Masaldan et al., 2019).

Due to the existence of the blood–brain barrier (BBB), the entry of metal ions into the brain is precisely regulated, mainly through a series of metal metabolism-related proteins. TfR1 is highly expressed on brain capillary endothelial cells of the BBB. It binds to circulating transferrin to form a Tf-Fe^3+^ complex, which enters the cell through receptor-mediated endocytosis. This complex is transported to the cytoplasm of brain cells either directly through astrocytes or via DMT1. Subsequently, ferrous iron can be utilized, stored in ferritin, or released from the cell at the abluminal side into the cerebrospinal fluid (CSF). After releasing iron ions, apo TF enters the bloodstream through arachnoid villi (Chiou et al., 2019). The BBB forms a formidable defensive wall that protects the brain against foreign molecules and separates the iron homeostasis of the brain from that of the periphery. This mechanism maintains an approximately saturated and stable state of iron in the brain, thereby sustaining the normal physiological functions of the nervous system (Belaidi and Bush, 2016; Jiang et al., 2017; Masaldan et al., 2019). Iron overload induces BBB breakdown, brain iron accumulation, brain mitochondrial dysfunction, impaired brain mitochondrial dynamics, tau hyperphosphorylation, Aβ accumulation, and decreased dendritic spines, which in turn trigger a range of neurotoxic effects (Sripetchwandee et al., 2016; Zeng et al., 2021; Dusek et al., 2022).

IRPs indirectly affect the function of the BBB and the distribution of iron in the brain by finely regulating iron homeostasis, which is of great significance for the occurrence and development of neurodegenerative diseases (Meyron-Holtz et al., 2004; Cassidy et al., 2020). There are currently no cures for neurodegenerative diseases; however, researchers have found that iron metabolism disorders are some of the mechanisms underlying their pathogenesis (Yan and Zhang, 2019).

#### Disorder of iron homeostasis and neuronal apoptosis

When iron homeostasis is disrupted, the metabolism and function of neurons are severely affected. Iron overload increases the amount of free iron within cells, generating a large amount of reactive oxygen species (ROS); triggering oxidative stress; damaging cell membranes, proteins, and DNA; and leading to neuronal death. It also interferes with cellular energy metabolism, causing issues such as glucose metabolism disorders and insulin resistance that affect the normal function of neurons (Wu et al., 2023; Levi et al., 2024; Zeidan et al., 2024). Conversely, iron deficiency may affect the development and synapse formation of neurons, as well as the synthesis of certain neurotransmitters, further impairing neuronal function (Matak et al., 2016; Levi et al., 2024).

The excessive accumulation of iron is an important factor leading to neuronal apoptosis and can induce neurotoxicity through different mechanisms. It can increase the permeability of the BBB, induce inflammation, affect the redistribution of iron in the brain, and thus alter brain iron metabolism (Sousa et al., 2020). Iron overload can generate ROS through the Fenton and Haber Weiss chemical reaction. When ROS levels exceed the antioxidant capacity of organelles, they can attack various biomolecules inside the cell, leading to cellular dysfunction and death (Wessling-Resnick, 2010; Zhang et al., 2022). In addition, excessive iron can cause mitochondrial dysfunction, leading to energy metabolism disorders, promoting the loss of mitochondrial membrane potential, and initiating the apoptotic pathway (Wessling-Resnick, 2010; Zhang et al., 2022). An iron homeostasis imbalance also promotes cell apoptosis by activating specific signaling pathways. For example, the p53 signaling pathway plays an important role in the cell apoptosis caused by iron overload. Research has shown that the upregulation of p53 can lead to changes in the expression of downstream genes involved in cell cycle arrest and the initiation of apoptosis programs, ultimately resulting in neuronal death (Wessling-Resnick, 2010; Ru et al., 2024). Iron accumulation can also activate endogenous apoptotic pathways, leading to the release cytochrome c through mitochondria to guide the formation of apoptotic bodies and further enhancing the transmission of apoptotic signals (Long et al., 2023; Vechalapu et al., 2024).

Disruptions in iron metabolism also participate in inflammatory reactions. Iron deficiency and excess can affect the inflammatory state of the body. Iron overload is often accompanied by an increased inflammatory response, and inflammatory factors such as tumor necrosis factor alpha and interleukin (IL)-6 can further promote neuronal apoptosis (Ni et al., 2022).

### Iron homeostasis in Alzheimer’s disease

AD is the most common type of senile dementia (Kenkhuis et al., 2021; Jin et al., 2024; Sun et al., 2024). AD is a progressive neurodegenerative disorder characterized by memory loss, cognitive dysfunction, and the deterioration of emotional and psychological symptoms (Paulsen et al., 2000; Tractenberg et al., 2002). Its pathological features include senile plaques, neurofibrillary tangles, elevated iron levels in the brain, neuroinflammation, and neuronal death (Kenkhuis et al., 2021; Khan et al., 2023; Mezzanotte and Stanga, 2024).

#### Effect of disordered iron metabolism on Alzheimer’s disease

Studies have identified a considerable correlation between excessive dietary iron intake and cognitive decline in mouse models, underscoring the critical role of iron metabolism in the pathogenesis of AD. Furthermore, research on patients with AD has revealed that iron deposition predominantly occurs in brain regions integral to motor control and cognitive function. These findings collectively highlight the importance of understanding iron’s impact on neurological health and its potential contribution to the development and progression of AD (Wang et al., 2019a; Peng et al., 2021; Pal et al., 2022; **Table 1**).

**Table 1:** Characteristics of iron homeostasis dysregulation in different neurodegenerative diseases

Disease	Pathological features	Key pathways involved
Alzheimer’s disease	Senile plaques, neurofibrillary tangles, cerebral iron deposition	IRPs-IRE, Fe-S cluster, ferroptosis
Parkinson’s disease	Loss of alpha synuclein, α-synuclein	IRPs-IRE, ferroptosis, mitochondrial dysfunction
Freudian ataxia	Frataxin deficiency, disorders of iron metabolism, oxidative stress	IRP1-IRE, Frataxin, Fe-S cluster, ferroptosis
Huntington’s disease	Mutant huntingtin, gene transcription abnormalities, oxidative stress, mitochondrial dysfunction	IRP1-IRE, STAT5-IRP1, ferroptosis
Amyotrophic lateral sclerosis	Gene mutations, oxidative stress, neuroinflammation, mitochondrial dysfunction, and metal toxicity	IRPs-IRE, neurotransmitter
Multiple sclerosis	Chronic inflammation and cerebral iron deposition	IRP1/2-IRE, oxidative stress
Neuroferritinopathy	L-ferritin gene mutation	IRP1/2-IRE, oxidative stress

IRE: Iron responsive element; IRPs: iron regulatory proteins.

Aβ is a fragment of amyloid precursor protein (APP) (Wilkins and Swerdlow, 2017; Li et al., 2025; Yao et al., 2025). As iron accumulates in the brain, the free radicals generated in the Aβ aggregation area can damage adjacent neurons, leading to a decline in cognitive and memory function. Tau is a microtubule-associated neuronal protein that accumulates in the somatic dendritic portion of neurons and forms a core component of neurofibrillary tangles, the second hallmark of AD, which exert neurotoxic effects (Johnson et al., 2023). Iron is associated with the aggregation, oligomerization, amyloidosis, and toxicity of Aβ (Masaldan et al., 2019). Fe^2+^ binds to the N-terminal region of Aβ and leads to Aβ modifications. Fe^2+^ also activates cyclin-dependent kinase5 and glycogen synthase kinase3β to promote tau phosphorylation (Guo et al., 2013; Wärmländer et al., 2019; Wang et al., 2023a; Tian et al., 2024). The resulting oxidative stress and metal toxicity of iron may lead to AD (Spotorno et al., 2020). Oxidative stress becomes more pronounced with increasing iron concentration, and the oxidation of proteins, lipids, and DNA in the Aβ aggregation region is more notable (Cho et al., 2010; Sadigh-Eteghad et al., 2015; Lee et al., 2020). Aβ is a core substance in the pathological characteristics of AD, and its accumulation not only leads to the formation of senile plaques but also produces neurotoxicity when Aβ binding to iron occurs, which further triggers oxidative stress and damages neurons (Cho et al., 2010; Sadigh-Eteghad et al., 2015). The 5′-UTR of APP contains an IRE-like structure, which implies APP has a potential function in the regulation of iron homeostasis and is specifically regulated by IRPs (Rogers et al., 2008; Cho et al., 2010; Khan et al., 2023). When intracellular iron levels are elevated, the expression of iron regulatory proteins (particularly IRP2) is increased, and the binding of IRPs to APP mRNA is attenuated, facilitating APP translation, which increases the production of Aβ (Berry et al., 2020; Wu et al., 2023). High concentrations of iron in cells promote APP cleavage through the amyloidosis pathway, and the deposition of Aβ_1–42_ fragments produced by cleavage can cause cell damage and death (Bandyopadhyay and Rogers, 2014; Everett et al., 2014; Prasanna and Jing, 2021). Due to the continuous cleavage of APP and the excessive phosphorylation of tau in the AD brain, the outflow of iron from neurons is hindered (Tsatsanis et al., 2020). The accumulation of iron ions leads to the hyperphosphorylation of tau protein, and the aggregation of hyperphosphorylated tau protein induces the accumulation of neuronal iron and aggravates neurofibrillary tangles. Tau hyperphosphorylation promotes the deposition of iron ions, and a vicious circle forms between the iron imbalance and Aβ/tau protein abnormalities, ultimately promoting the development of AD (Wu et al., 2023).

#### Iron homeostasis regulation in Alzheimer’s disease

The disruption of iron metabolism and changes in the expression of IRPs in iron metabolism pathways can lead to the accumulation of iron in the brain and induce oxidative stress, resulting in neuronal damage. IRPs/IREs different from those of normal people were found in the brains of patients with AD, indicating that the regulatory function of IRPs is abnormal in AD and affects the expression of iron metabolism-related genes (Piñero et al., 2000b). Through testing, it was found that the levels of antioxidant stress in the hippocampus and amygdala were significantly higher than those in other regions. Iron accumulation and oxidative stress can lead to an increase in IRP1 activity, which in turn regulates TfR1 to enhance iron absorption. By reducing the concentrations of ferritin H and ferritin L, intracellular free iron levels are increased, and positive feedback mechanisms regulate oxidative stress in cells, forming a vicious cycle (Piñero et al., 2000b; Khan et al., 2023). In patients with AD, FPN was decreased in the brain and peripheral serum iron levels were low, which are consistent with the high level of IRP1 and the impairment of cellular iron export. By observing mouse experiments, researchers found FPN1 deficiency can lead to hippocampal atrophy and cognitive impairment (Bao et al., 2021). However, studies have also shown that the concentrations of ferritin heavy chain and ferritin light chain are elevated in the hippocampus of patients with AD, at levels three times higher than those in normal human brains (Ashraf et al., 2020). IRP1 can also decrease the mRNA stability of hypoxia inducible factor 2α and cause other metal ion deficiencies (Berry et al., 2020). IRP2 plays a more important role in the regulation of iron homeostasis in AD; it can indirectly inhibit the deposition of Aβ by reducing the expression of APP and lead to pathological changes to tau proteins in neurons when iron homeostasis is abnormal, thus reducing neuronal damage (Li et al., 2022; **Table 1**).

### Iron homeostasis in Parkinson’s disease

The prevalence of PD, the second most common neurodegenerative disorder, continues to rise as the population ages (Berry and Moustafa, 2023). Disability and death from PD are increasing rapidly worldwide, yet there is no cure. Clinically, males outnumber females, and cases are characterized by motor dysfunction, rigidity, and bradykinesia, with cognitive decline and dementia occurring in the advanced stages of the disease (Caligiore et al., 2016; Poewe et al., 2017). The main pathological features of PD are a loss of dopaminergic neurons in the substantia nigra (SN), a lack of dopamine in the striatum, and the accumulation of α-syn inclusions (Xu et al., 2018; Berry and Moustafa, 2023). More and more studies are showing that the disorder of iron homeostasis is a key factor leading to these neurodegenerative changes (Mounsey and Teismann, 2012; Ma et al., 2021; **Table 1**).

#### Effect of iron metabolism disorder on Parkinson’s disease

Patients with PD have abnormally elevated levels of iron in their brains, which reflects the dysfunction of the brain’s iron homeostasis. Disruptions to iron metabolism in SN is a common feature of PD, and in the brains of patients with PD, the iron levels of individual substantia nigra dopaminergic neurons can be upregulated by nearly two-fold (Oakley et al., 2007). An imbalance in iron metabolism is closely related to dopamine metabolism and mitochondrial dysfunction, which may jointly participate in the pathogenesis of PD (Buoso et al., 2024). Research has also demonstrated that disordered iron homeostasis not only exists in PD brain tissue but also increases the content of ferritin in the CSF compared to that of normal individuals (Liu et al., 2017). This also proves that the BBB is disrupted. In patients with PD, abnormal iron metabolism, as well as an increase in inflammatory factors, is seen in the CSF. It is believed that the iron content of the CSF increases with the increase in inflammatory factors (Hu et al., 2015). Nitric oxide (NO) is considered an upstream pathogenic factor in the pathogenesis of PD, as it is involved in the accumulation of substantia nigra iron and degeneration of dopaminergic neurons. NO can inhibit APP translation and thereby exacerbate iron retention in dopaminergic neurons (Hu et al., 2015). Iron levels are positively correlated with the levels of NO and IL-1β in the CSF. Increased amounts of iron in the brain may lead to the sustained activation of microglia and the production of excess inflammatory factors, resulting in neuronal death and sleep behavior disorder symptoms in patients with PD (Hu et al., 2015). Chronic inflammatory responses can lead to metabolic disorders of iron, and inflammatory cell activation may promote the release of iron, resulting in an increase in the local iron concentration and causing damage to neurons.

Some authors have found, through Mendelian randomization studies, that peripheral iron may also affect PD. The liver and spleen play a crucial role in iron metabolism, and the liver iron content predicted by genetics is positively correlated with the risk of PD (Yang et al., 2024). However, further explorations are needed into how peripheral iron metabolism affects the central nervous system and plays a role in the pathogenesis of PD.

#### Iron homeostasis regulation in Parkinson’s disease

IRPs are key factors in the regulation of iron homeostasis and play important roles in the imbalance of iron metabolism in PD. In patients with PD, IRP1 maintains iron homeostasis by regulating key iron transporters (Salazar et al., 2008; Urrutia et al., 2017; Costain et al., 2019). The activation of IRP1 triggers the upregulation of DMT1 and TfR1 and the downregulation of FPN, resulting in increased iron uptake and the accumulation of LIP (Lee et al., 2009; Song et al., 2010; Mena et al., 2011; Aguirre et al., 2012; Urrutia et al., 2017; Xu et al., 2018). These regulatory processes have been demonstrated in animal models of PD, such as those induced by MPTP and 6-OHDA, suggesting that IRP1 contributes greatly to the imbalance in iron metabolism (Salazar et al., 2008; Jiang et al., 2010; Zhang et al., 2013). In animal models, IRP1 plays a central role in the imbalance of iron metabolism in SN, which is accompanied by a loss of dopaminergic neurons in the substantia nigra and striatum (Carroll et al., 2011; Dexter et al., 2011). IRP1 also affects the function of mitochondria through its regulation of iron metabolism, and inhibiting the formation of iron-sulfur clusters in mitochondria leads to the accumulation of iron. The activation of IRP1 affects the function of iron regulatory proteins and the tricarboxylic acid cycle, and aggravates pathological changes related to PD (Shi et al., 2010; Zhang et al., 2014; Berry and Moustafa, 2023).

Iron deposition in PD induces the aggregation of α-syn (Chen et al., 2019), which is a neuropathological marker of PD that aggregates abnormally in the substantia nigra to form Lewy bodies (Praschberger et al., 2023; Wang et al., 2024). The 5′-UTR of α-syn contains an IRE-like structure that can bind to IRPs to upregulate DMT1 and downregulate FPN, thereby affecting iron metabolism (Febbraro et al., 2012; Zhou and Tan, 2017; Ma et al., 2021; Mi et al., 2021). This interaction further aggravates the iron metabolic imbalance in PD, and IRPs directly affect α-syn aggregation and toxicity by regulating these iron transporters (Salazar et al., 2008; Mounsey and Teismann, 2012). α-Syn silencing results in the accumulation of TfR1 complexes in reclaimed endosomes, and α-syn overexpression increases TfR1 expression and ferritin levels, possibly in response to excess brain iron, through potential feedback loops to accelerate neuronal ferroptosis, leading to cognitive decline and proteinopathy (Ma et al., 2021). There is a direct correlation between ferritin and α-syn. The downregulation of α-syn reduces ferritin, while its overexpression leads to ferritin accumulation. The overexpression of α-syn damages ferritin degradation and iron release by impairing lysosomal function, a process known as ferritin autophagy, which may explain the retinal degeneration observed in PD cases (Baksi and Singh, 2017).

IRP2 also plays an important role in the regulation of iron homeostasis in PD. Studies have shown that the overexpression of IRP2 is highly likely to cause a decrease in motor ability, abnormal gait, and iron accumulation in substantia nigra in patients with PD, forming a vicious circle (Regan et al., 2008). It was found that the massive accumulation of iron in SN following IRP2 gene deletion preceded the onset of dopaminergic neuronal degeneration and PD-like symptoms, which were accompanied by the deposition of ubiquitin-positive protein aggregates and inclusion bodies in the mouse brain (Zhou and Tan, 2017). These alterations exacerbate neuronal apoptosis and PD symptoms, which in turn lead to an increase in iron content in astrocytes around neurons, ultimately creating a vicious cycle that promotes PD progression (Ci et al., 2020; **Table 1**).

### Iron homeostasis in Friedreich’s ataxia

FRDA is a rare genetic neuromuscular disease. It is an autosomal recessive neurodegenerative disorder caused by a deficiency of the frataxin (FXN) protein and is the most common inherited ataxia (Krasilnikova et al., 2023). The clinical features of FRDA include progressive gait instability, limb ataxia, cardiomyopathy, and diabetes mellitus. The disease typically manifests in adolescence (Zhao et al., 2020). Although the clinical phenotype of FRDA is highly variable, it usually involves considerable motor dysfunction. In the early stages, patients may exhibit only mild motor impairment, but symptoms progressively worsen over time, leading to severe physical disability. FXN plays a key role in the biosynthesis of the Fe-S clusters, and its deficiency leads to mitochondrial iron metabolism disorders, ultimately resulting in an imbalance in iron homeostasis and oxidative stress-related neuronal damage (Isaya, 2014; Cook and Giunti, 2017; Buesch and Zhang, 2022). Decreased mitochondrial function and an impaired electron transport chain lead to Fe-mediated production of ROS, which has been shown to correlate positively with the severity of FRDA (**Table 1**).

#### Effect of iron metabolism disorder on Friedreich’s ataxia

Research has shown that patients with FRDA have abnormally low levels of iron in their blood. This lack of iron may lead to the activation of the body’s high-affinity iron uptake mechanism, which manifests as an increase in the expression of high-affinity iron uptake proteins (Gordon, 2000). The accumulation of iron in the brain and myocardium of FRDA can be visualized through MRI and histological staining (Schipper, 2012), and research suggests that, while the cells of patients with FA have an increased demand for iron, they are unable to effectively utilize this iron.

The biochemical properties of the FXN protein include its abilities to bind iron, provide iron to other iron-binding proteins, oligomerize, store iron, and control iron redox chemistry. Its primary function is as an iron-binding chaperone in Fe-S cluster synthesis (Isaya, 2014). A reduction in FXN expression leads to a decrease in the activity of Fe-S cluster proteins (such as aconitase and succinate dehydrogenase), the accumulation of mitochondrial iron, a decrease in functional iron in the cytoplasm, and the impairment of cellular iron homeostasis (Ye and Rouault, 2010; Dusek et al., 2022).

#### Iron homeostasis regulation in Friedreich’s ataxia

A reduction in FXN expression not only disrupts mitochondrial function, but also activates IRPs, further exacerbating the imbalance in iron homeostasis (Campuzano et al., 1996; Tong and Rouault, 2006). As Fe-S cluster synthesis is blocked, IRP1 changes from its active aconitase form to an IRE-binding protein with RNA-binding ability, resulting in more iron uptake by cells and its deposition in mitochondria (Li et al., 2008; Levi et al., 2024). This vicious cycle leads to further impairments in mitochondrial function and promotes oxidative stress and neuronal degeneration (Tong and Rouault, 2006). The expression of IRP1 is decreased in FRDA, as confirmed in FRDA model mice (KIKO mice). Long-term exercise can partially restore IRP1 levels, thus mitochondrial function is improved and oxidative stress is reduced, preventing the occurrence of FRDA symptoms (Zhao et al., 2020).

An FXN deficiency is closely associated with oxidative stress; however, earlier studies indicated that FRDA is not solely dependent on oxidative damage. For instance, research conducted by Seznec et al. (2005) demonstrated that, even in the absence of significant oxidative stress, the iron-sulfur cluster synthesis function of IRP1 was impaired. Concurrently, its activity as an IRE-binding protein was found to be increased. These findings suggest that the abnormal activity of IRP1 is not exclusively linked to oxidative stress. Instead, it may contribute to the progression of FRDA through mechanisms that operate independently of oxidative stress.

Elevated levels of TfR1 and ferritin were observed in the peripheral blood mononuclear cells of patients with FRDA, indicating there were changes in iron transport and storage mechanisms. Although the mRNA expression level of TfR was low, the steady-state level of TfR1 increased in iron-loaded FRDA fibroblasts, further indicating the presence of regulatory abnormalities in iron metabolism (Pantopoulos, 2021; **Table 1**).

### Iron homeostasis in Huntington’s disease

HD is an autosomal dominant neurodegenerative disorder characterized by neuronal degeneration and cell death due to the presence of a mutant huntingtin gene (HTT). Mutant huntingtin (mHTT) contains too many glutamine residues, and this abnormal protein gradually accumulates in neurons, causing cell dysfunction and eventually triggering the death of nerve cells. The main clinical signs of HD form a triad of dyskinesia, cognitive decline, and psychiatric symptoms (Ajitkumar and De Jesus, 2025). The disease usually begins in middle age, progresses slowly, and eventually leads to the death of the patient. Occasionally, HD may begin in adolescence, and this early-onset form of HD typically presents with more severe symptoms (Kim et al., 2021). Pathologically, the main feature of HD is the degeneration of spiny neurons in the striatum region of the brain, resulting in the gradual death of nerve cells. mHTT not only induces neuronal dysfunction but also results in a variety of deleterious pathological effects, including abnormal gene transcription, oxidative stress, mitochondrial dysfunction, and alterations in intracellular calcium homeostasis (Lim et al., 2008; **Table 1**).

#### Effect of iron metabolism disorder on Huntington’s disease

Iron accumulation occurs in the basal ganglia region of patients with HD (Sánchez-Castañeda et al., 2015). In HD, mHTT exacerbates intracellular iron metabolic imbalances through multiple mechanisms. Studies have shown that the mHTT protein can increase the influx of iron into nerve cells, which leads to iron accumulation and exacerbates oxidative stress, further damaging neurons (Yao et al., 2024a). This oxidative stress mainly originates from iron overload because excessive iron leads to mitochondrial dysfunction and the degeneration of nerve cells by promoting the production of free radicals (Agrawal et al., 2018; Wang et al., 2023b). Therefore, the dysregulation of iron homeostasis is an important driver of HD neurodegeneration. The increase in iron is not only a result of pathological changes but may also play a promotional role in disease progression.

In the early stages of HD, there is a significant increase in iron content in the subcortical structure and surrounding white matter. This phenomenon suggests that changes in iron homeostasis may be one of the early signs of the disease, existing even before clinical symptoms appear. This early accumulation of iron may lay the foundation for subsequent neurodegenerative changes (Johnson et al., 2021). Researchers demonstrated that increasing the dietary iron intake of newborn R6/2 HD mice enhanced the development of HD and promoted oxidative stress and energy dysfunction in the brain (Berggren et al., 2015). The excessive accumulation of iron may lead to oxidative stress, resulting in neuronal apoptosis and dysfunction. Iron-induced oxidative stress is considered one of the most important factors triggering neurodegenerative changes in HD (van de Zande et al., 2023).

In HD, increased iron deposition is not limited to neurons, as it also involves oligodendrocytes, which are among the most iron-rich cells in the brain. Myelinolysis in HD may induce a compensatory response by oligodendrocytes, resulting in the uptake and accumulation of more iron by these cells (Yao et al., 2024a). This compensatory increase in iron deposition may trigger toxic effects, further promote demyelination, and exacerbate neuronal damage. Because of the important role of oligodendrocytes in maintaining myelin integrity in the central nervous system, their dysfunction may accelerate the progression of neurodegeneration in HD.

#### Iron homeostasis regulation in Huntington’s disease

mHTT is an iron-regulation protein that further enhances iron accumulation in the brain by altering the expression of intracellular proteins associated with iron metabolism (Wang et al., 2023b). IRPs play an important role in this process, especially IRP1, which acts as a key regulator of iron metabolism and regulates iron homeostasis through its interaction with huntingtin mRNA. It has been shown that the 5′-UTR of HTT mRNA contains an atypical IRE capable of binding to IRP1 (Chen et al., 2021b; Pfalzer et al., 2022). This interaction may enhance iron uptake by increasing the expression of TfR1, thereby aggravating iron accumulation in the brain and exacerbating oxidative stress and neuronal damage (Wang et al., 2023b). Niu et al. (2018) showed that mHTT promoted iron deposition in the striatum by upregulating the expression of IRP1 and Tf in a mouse model of HD. mHTT regulates IRP1-mediated iron uptake, resulting in a further imbalance of iron homeostasis, making neurons more vulnerable to oxidative stress, and thus aggravating the pathological progression of HD. However, other studies reported the decreased expression level of IRP1 in HD model mice, which led to a decrease in TfR1 and increase in FPN expression, which may have been compensatory responses to the increase in intracellular LIP (Chen et al., 2013). This phenomenon suggests that HD neurons have a self-regulatory mechanism that allows them to cope with iron overload and restore iron homeostasis by reducing iron uptake and increasing iron output.

In addition to the direct effects of mHTT on IRP1, the transcription factor STAT5 is also involved in the regulation of iron homeostasis in HD. STAT5 was found to further promote iron accumulation in the brain by upregulating the expression of IRP1, leading to increased levels of ferritin and TfR1 (Niu et al., 2024). Activation of this transcription factor-IRP1 axis may be another important iron metabolism disorder mechanism in HD. Iron overload not only aggravates oxidative stress but may also further aggravate neuronal damage in HD by affecting other iron metabolic pathways (**Table 1**).

### Iron homeostasis in amyotrophic lateral sclerosis

ALS is a fatal neurodegenerative disease of the central nervous system whose early symptoms are difficult to recognize. The incidence of this disease is higher in individuals over 60 years old, and it is relatively more common in female patients (Feldman et al., 2022). ALS primarily affects motor neuron degeneration and death in the cerebral cortex, spinal cord, and brainstem, which in turn causes progressive muscle atrophy, affecting limb movement, swallowing (causing dysphagia), speech (causing dysarthria), and respiratory function, and resulting in the progressive weakening of voluntary skeletal muscles (Sripetchwandee et al., 2016; Feldman et al., 2022). The pathogenesis is complex, including genetic mutations, oxidative stress, neuroinflammation, mitochondrial dysfunction, and metal toxicity (Wood and Langford, 2014; **Table 1**). In recent years, studies have shown that iron levels are significantly increased in the spinal cord of patients with ALS, and secondary iron accumulation occurs in their microglia. There is a close association between the disruption of iron homeostasis and the degeneration of motor neurons in this condition (Feldman et al., 2022).

C9ORF72 is one of the pathogenic genes of ALS, and amplification of its heavy non-coding repeat is the most common mutation in ALS (Smeyers et al., 2021). Studies have predicted the presence of IRE in the 5′-UTR of the mRNA encoding C9ORF72, which may explain the findings of elevated iron and ferritin levels in patients with ALS. Therefore, there may be a potential mechanistic link between iron and the iron-modulated translation of C9ORF72 (Zhou and Tan, 2017; Smeyers et al., 2021). In addition, multiple studies have found that serum iron and ferritin levels are significantly elevated in patients with ALS. Serum ferritin is a protein that stores iron, and its level is positively correlated with the duration of the disease, indicating that, as the disease progresses, iron accumulation may worsen the condition (Su et al., 2013). The increase in iron deposition is partially related to the increased expression of TFR1 in glial cells. Although the expression of FPN in ALS increases, it is still lower than that of DMT1, which means that iron elimination is less than iron inflow, which is conducive to iron deposition (Jeong et al., 2009).

The mechanisms of action of IRPs in ALS may explain their roles in other neurodegenerative diseases, such as AD and PD. These disorders are characterized by the dysregulation of iron homeostasis and an increase in oxidative stress. As key regulators of iron metabolism, IRPs may play pivotal roles in the pathological processes common to these conditions (Zumbrennen-Bullough et al., 2014). Consequently, an in-depth investigation into the roles of IRPs in ALS could not only elucidate the pathogenesis of ALS but also offer novel insights leading to the development of treatments for other neurodegenerative diseases (**Table 1**).

### Iron homeostasis in multiple sclerosis

MS is the most common chronic, inflammatory, demyelinating, neurodegenerative disease of the central nervous system (Van San et al., 2023). The pathologic hallmark of MS is the presence of diffuse “sclerosing plaques” in multiple areas of the central nervous system, and its pathophysiology includes inflammatory demyelination, neuronal injury, and brain injury (The Lancet Neurology, 2021; **Table 1**).

There is an association between cerebral iron deposition and normal aging, as well as MS. MRI studies have shown excessive iron deposits form in the gray matter of patients with MS, mainly concentrated in the basal ganglia and white matter, especially near lesion areas, and this abnormal iron accumulation may be related to disease progression (Stüber et al., 2016; Hamdy et al., 2022). MS lesions exhibit heterogeneity in their iron accumulation patterns. In some lesions, the concentration of iron significantly increases, while in other lesions, it may be relatively low. These differences may be related to such factors as the activity and type of lesion, as well as the age of the patients (Hametner et al., 2013; Popescu et al., 2017). The abnormal deposition of iron may be associated with the development of MS and clinical manifestations that lead to oxidative stress, which further damages brain cells. Iron is primarily stored in myelin and oligodendrocytes. In demyelinating lesions, the release of iron may exacerbate tissue damage and further accelerate neurodegeneration (Hametner et al., 2018). It has been suggested that iron deficiency can also affect the pathology of MS, as iron deficiency can affect the health and regeneration of oligodendrocytes and myelin sheaths (Patel et al., 2022). In patients with MS, serum iron levels may show a decreasing trend that may be related to their overall health status and disease activity (Tang et al., 2024; **Table 1**).

In this context, it has been established that IRP2, one of the genes associated with iron metabolism in MS, is also involved in cellular iron homeostasis and iron ion transport (Tang et al., 2024).

Transferrin levels are associated with an increased risk of MS, while transferrin saturation is associated with a reduced risk of MS (Tang et al., 2024). Increased transferrin concentrations are a sign of iron deficiency. When iron levels are low, transferrin concentrations usually increase to regulate iron homeostasis in the blood. An increase in transferrin saturation indicates an increase in the level of iron in the blood. Transferrin’s roles include the transport and metabolism of iron, and its activity in central nervous system iron transport is regulated by IRPs. Transferrin may be involved in regulating iron utilization and storage within oligodendrocytes. Iron is essential for myelination, and oligodendrocytes are the main producers of myelin (Tang et al., 2024). Therefore, it can be assumed that IRPs’ roles in MS are similar to the roles they have in the above disorders.

### Iron homeostasis in neuroferritinopathy

NF is a rare neurodegenerative disease characterized by the gradual accumulation of iron in the brain. This disease is caused by mutations in the L-ferritin gene (FTL) and is the result of autosomal dominant inheritance (**Table 1**). The occurrence of this disease is related to an imbalance of iron metabolism leading to the excessive accumulation of iron in nerve cells. It mainly affects the basal ganglia in the brain that control movement, leading to a series of motor disorders and other neurological symptoms. NF typically manifests as progressive movement disorders, cognitive decline, and some other neurological symptoms such as emotional changes and sleep disorders (Marchand et al., 2022). NF patients’ brains exhibit a significant ongoing increase in iron content, leading to severe neurological damage (Chinnery et al., 2006; Marchand et al., 2022). Despite the increase in brain iron levels, many patients with NF have normal or low serum ferritin levels. This phenomenon contrasts sharply with the abnormal storage of iron in the brain, indicating significant differences in the distribution of iron between different tissues (Levi and Rovida, 2015).

The pathogenesis of NF is primarily attributed to mutations in the coding region of the IRE regulatory gene, i.e., mutations at the C-terminus of FTL (Zhou and Tan, 2017). The 460InsA mutation is the most common type and involves adenine insertions at positions 460–461 (Kumar et al., 2016). In addition, there are some rare mutations that cause a dysregulation of iron metabolism, making it difficult for the body to effectively regulate iron levels in the brain (Zhang et al., 2021). This genetic mutation disrupts the normal function of the FTL. Consequently, the mutated FTL gene results in a protein with impaired stability and a reduced ability to properly retain deposited iron, leading to a range of clinical manifestations (Muhoberac and Vidal, 2019; Marchand et al., 2022).

The primary function of cytosolic ferritin is to maintain a balance between the cytosolic LIP, which includes redox-active forms of iron necessary for cytosolic and mitochondrial enzyme functions, and excess iron, which can induce the formation of ROS and cause considerable cellular damage. Ferritin regulation is primarily governed by cytosolic iron levels and, at the translational level, by the IRE/IRP mechanism. In patients with NF, fibroblasts exhibit elevated ferritin levels, decreased TfR1, and increased total iron and ROS levels. This dysregulation of iron homeostasis and the resultant oxidative stress are considered the main contributors to cell death in NF (Cozzi et al., 2021). NF is a chronic progressive disease with no specific treatments, so the prognosis is relatively poor. Further research on the relationship between IRPs’ regulation of iron homeostasis and NF may provide new ideas for the treatment of this disease (**Table 1**).

### Ferroptosis and neurodegenerative diseases

Ferroptosis is a recently described form of iron-dependent cell death that is accompanied by a series of changes in cell morphology, metabolism, and protein expression. The metabolic disruption of lipid peroxides is catalyzed by iron and leads to the inactivation of glutathione peroxidase 4 (GPX4), which triggers cell death (Zhang et al., 2022). One of the core features of ferroptosis is its dependence on iron, which distinguishes it from other forms of cell death. Another important characteristic is the accumulation of lipid peroxidation, which, once it reaches a certain level, can lead to membrane rupture and cell death.

Ferroptosis involves characteristic changes in cellular morphology, including mitochondrial atrophy, a reduction or disappearance of the inner mitochondrial membrane spine, an increase in mitochondrial membrane density, and rupture of the outer membrane (Zhang et al., 2022). In terms of biochemistry, the process is mainly initiated by a depletion of glutathione, resulting in a decrease in the activity of GPX4, which then produces ROS and promotes ferroptosis. In immunology, its involvement in the release of pro-inflammatory mediators may trigger neuroinflammation, further exacerbating neuronal damage and accelerating the progression of the disease (Turchi et al., 2020). Genetic studies indicate there are changes in the expression of genes that regulate iron homeostasis and lipid peroxidation metabolism (Gao et al., 2019).

An imbalance between the import, storage, and export of iron may affect the susceptibility of cells to iron death. For example, the increased degradation of ferritin can increase the LIP and enhance ferroptosis (Mancias et al., 2014); while excessive amounts of iron can cause the peroxidation of cell membrane lipids, leading to cellular dysfunction and death (Pal et al., 2022). Brain tissue is particularly susceptible to oxidative stress, and increased oxidative stress is a characteristic of several neurodegenerative diseases. We have discussed in detail how iron homeostasis disorders present in six neurodegenerative diseases in previous sections, and the known mechanisms of ferroptosis are detailed below.

GPX4 mRNA and protein levels were shown to be abnormally increased in an AD mouse model (da Rocha et al., 2018). The biosynthesis of GPX4 requires glutathione (GSH) (da Rocha et al., 2018), the expression of which is also reduced in the cortex, a phenomenon associated with cognitive decline (Karelson et al., 2001). In an examination of GPX4-knockout mice, the mice exhibited neuronal necrosis, which became more severe when there was a lack of vitamin E—an iron death inhibitor—in the diet. Studies have found that most proteins involved in ferroptosis are regulated by Nrf2. The levels of Nrf2 in the AD brain are significantly lower than those in the normal brain, which may be one of the reasons for the occurrence of ferroptosis in this condition (Osama et al., 2020). Iron interacts with Aβ and tau to induce ROS levels, leading to ferroptosis (Derry et al., 2020). In contrast, inhibiting ferroptosis can effectively alleviate the symptoms of AD, indicating that ferroptosis increases the severity AD (Cozza et al., 2017; Li et al., 2020; Chen et al., 2021a).

IRP2 induces iron death by regulating the p53-SLC7A11-ALOX12 pathway, which is particularly important in PD. By binding to the IRE structure in p53 mRNA, IRP2 post-transcriptionally upregulates p53 expression, resulting in the downregulation of SLC7A11 and GPX4, increasing lipid peroxidation levels, inducing iron death, and further exacerbating PD pathology (Chang et al., 2023; Li et al., 2024). The depletion of GSH and GPX4 in the SN pars compacta may promote the progression of PD by regulating ferroptosis (Ding et al., 2023).

In HD, mHTT is cleaved into toxic fragments that form monomers or small oligomers within neurons. These toxic fragments then inhibit proteasomes and autophagy, leading to the abnormal accumulation of misfolded proteins and mitochondrial dysfunction. Eventually, excessive ROS, extensive lipid peroxidation, and iron deposition together contribute to the occurrence of ferroptosis.

**Figure 4:**
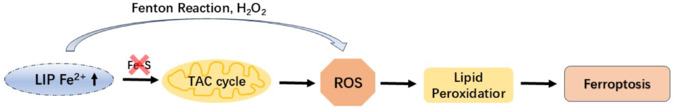
Iron accumulation induces ferroptosis. The abnormal increase in divalent iron ions in the labile iron pool (LIP) triggers the Fenton reaction, leading to the accumulation of H_2_O_2_ and impairment of Fe‒S cluster synthesis. This results in abnormal mitochondrial energy metabolism, the production of reactive oxygen species (ROS), and further induction of lipid peroxidation reactions, ultimately causing ferroptosis.

In patients with FRDA, FXN is reduced in the blood and fibroblasts, and this is accompanied by an increase in protein glutathionylation. All these defects may render FRDA cells susceptible to ferroptosis, which is a key mechanism of neurodegeneration in FRDA (Petrillo et al., 2019; La Rosa et al., 2020). The deficiency of FXN leads to disordered iron metabolism within cells. With the accumulation of iron, the level of oxidative stress rises, promoting the occurrence of ferroptosis. This has been confirmed in various tissues of patients with FRDA, as well as in cellular and animal models. When the level of FXN is too low, the function of iron-sulfur proteins is impaired, and the activities of aconitase and respiratory chain complexes I, II, and III are reduced. Subsequently, defects in the activity of iron-sulfur proteins in mitochondria and other cellular compartments lead to an abundance of ROS. Moreover, the oxidation of iron in mitochondria may be harmful because it generates toxic free radicals (Pandolfo, 2012; Levi et al., 2024). This ultimately leads to the over-production of ROS from the Fenton reaction, which is exacerbated by the fact that the antioxidant system appears to be compromised in FRDA (Cotticelli et al., 2019; Du et al., 2020; La Rosa et al., 2021). In patients with FRDA, cellular ferroptosis is not limited to motor neurons; it also impacts other types of neurons, leading to widespread neurological dysfunction (Turchi et al., 2020; La Rosa et al., 2021; **Figure 4**).

## Discussion and Outlook

Maintaining systemic iron homeostasis is crucial for the normal function and survival of nerve cells, and its role in neurodegenerative diseases is increasingly attracting attention. The complexity of iron homeostasis lies in the fact that disordered iron metabolism not only affects the survival of neurons but also a variety of pathological processes. The excessive deposition and abnormal distribution of iron are important pathological features in neurodegenerative diseases. The deletion of IRP genes leads to a severe iron deficiency, which affects the normal development and function of neurons. IRPs may have an impact on the occurrence and progression of neurodegenerative diseases and may influence the pathological processes by regulating ferroid homeostasis. In addition, ferroptosis further exacerbates neuronal death by inducing lipid peroxidation and inhibiting the activity of GPX4, thereby deeply contributing to neurodegenerative diseases (Zhang et al., 2023). In summary, the regulation of iron homeostasis plays a crucial role in the pathogenesis of neurodegenerative diseases. Future research should further explore the complex relationships between iron metabolism and neurodegenerative diseases, as well as new therapeutic strategies for targeting iron homeostasis, with the aim of providing new directions and methods for the treatment of these diseases.

### Central role of iron metabolism in neurodegenerative diseases

Traditionally, the mechanisms of morbidity in neurodegenerative diseases, such as AD, PD, and HD, revolve around protein misfolding, aggregation, and synaptic dysfunction. However, there is growing evidence that the dysregulation of iron metabolism also plays a central role in these diseases. The imbalance in iron homeostasis appears not only to be a result of the diseases but also one of the driving forces behind their development.

IRPs are key iron regulatory proteins whose regulatory mechanisms are critical for maintaining the intracellular iron balance. In neurodegenerative diseases, IRPs exert regulatory roles that play a part in processes, such as oxidative stress, protein aggregation, the synthesis of neurotransmitters, mitochondrial dysfunction, and neuroinflammation, that aggravate neuronal injury and death. A large number of studies have also confirmed that related proteins, such as APP, α-syn, FXN, and mHTT, are involved in the regulation of cellular iron homeostasis by IRPs, and some proteins are, themselves, iron-modulating factors.

Recent studies have revealed the close relationship between iron metabolism and mitochondrial dysfunction. In patients with PD in particular, mitochondrial complex I dysfunction is highly correlated with excessive iron accumulation. IRPs directly affect mitochondrial function by regulating the balance of iron in the mitochondria. The excessive accumulation of iron not only leads to mitochondrial oxidative stress but also weakens the energy metabolism function of mitochondria, and ultimately leads to the death of neurons. This finding challenges the previous view of mitochondrial dysfunction as a feature of energy damage only, and emphasizes the complex interaction between iron metabolism and mitochondrial health.

The emergence of the concept of ferroptosis, an iron-dependent cell death mechanism, has further broadened our understanding of cell death mechanisms in neurodegenerative diseases. Traditionally, our understanding of the death of nerve cells has mainly focused on apoptotic and necrotic pathways; however, the role of ferroptosis implies iron has a unique role in the development of diseases. It has been found that IRP2 in particular can induce iron death by regulating the p53-SLC7A11-ALOX12 pathway and thereby exacerbate neuronal damage. This discovery has provided a new perspective of the mechanisms of cell death in neurodegenerative diseases, which is especially relevant for the intervention and reversal of these diseases.

These findings have significantly expanded our understanding of the pathophysiology of such diseases and suggest that the regulation of iron metabolism may be a novel therapeutic target.

### Future research directions and clinical applications

Based on the core role of iron metabolism in neurodegenerative diseases, future studies should further explore their specific regulatory mechanisms and clinical application prospects in different diseases. Current clinical research primarily focuses on the correlation between iron homeostasis and neurodegenerative diseases. By observing changes in iron levels within patients’ bodies, researchers hope to identify early markers of iron homeostasis disorders to allow interventions to be provided before the onset of disease. In basic research, scientists are exploring the mechanisms of iron’s action within cells, including how it affects cellular signaling pathways and gene expression. These studies will help us to understand how iron homeostasis disorders lead to neuronal dysfunction. Future research may move toward the design of personalized treatments. By understanding individual differences in iron metabolism, doctors can develop more precise treatment plans for patients, specifically regulating their iron levels to slow the progression of neurodegenerative diseases.

#### Drug development targeting iron metabolism

Iron chelators are a class of drugs that can bind to iron ions and promote their excretion. Because dysregulated iron homeostasis is prevalent in neurodegenerative diseases, iron chelators appear to improve the symptoms of each disease. Iron chelators can slow disease progression by regulating the activity of IRPs and reducing iron accumulation (Sohn et al., 2008). For example, iron chelators such as deferoxamine (DFO) and deferiprone can inhibit the expression of IRE-regulated APP and α-syn in brain tissue, reduce the production and deposition of Aβ, and thus improve the cognitive function of patients with AD (Ma et al., 2021). Currently, only deferiprone has entered the clinical trial phase, and its anti-inflammatory properties are reported to be beneficial for neurodegenerative diseases (Wang et al., 2022). In addition, iron chelators can reduce dyskinesia and neurodegenerative symptoms in patients with PD. The disadvantage of DFO is that it may lead to iron depletion and anemia, which are not ideal clinical outcomes (Devos et al., 2020).

The IRP-IRE signaling pathway is implicated to participate in the regulation of APP and α-syn translation, and the use of small-molecule IRE chemical inhibitors to reduce APP and α-syn levels and attenuate protein aggregation may have therapeutic implications for neurodegenerative diseases in humans. The APP gene in AD encodes a specific and fully functional IRE RNA stem-loop in the 5′-UTR that is regulated by IRPs. Chemical IRE inhibitors have been identified that reduce ferritin and Tf expression to attenuate the excess iron accumulation in the brain in AD or PD. Potent IRE inhibitors (e.g., Posiphen) are true APP 5′-UTR-directed translational blockers that reduce Aβ production in the human CSF (Rogers et al., 2011). Paroxetine is another IRE inhibitor that can modulate APP expression through its action on the APP transcript 5′-UTR (Zhou and Tan, 2017). Antioxidants also have potential application in the treatment of neurodegenerative diseases. For example, imidazolyl acetophenone oxime ester, which was initially screened as a hot new drug with the potential to effectively treat AD, and its derivatives have powerful inhibitory activity against NO production and effects on H_2_O_2_-induced cell damage and iron homeostasis imbalance (Teleanu et al., 2022).

Recently, evidence to suggest that food can be used to treat neurodegenerative diseases has emerged. Theoretically, a low-iron diet may help reduce the accumulation of iron in the brain, thereby lowering the risk of oxidative stress and ferroptosis and potentially playing a positive role in the prevention and alleviation of certain neurodegenerative diseases. The ketogenic diet has demonstrated potential therapeutic benefits for AD, PD, and ALS, leading to some symptoms showing improvement in relevant animal experiments (Tao et al., 2022). Emerging research suggests that the ketogenic diet exerts regulatory control over iron homeostasis by modulating key iron-related proteins. Specifically, the ketogenic diet has been shown to diminish the expression of TfR1 and DMT1, while concurrently enhancing the expression of FTH1 and FPN1. This intricate modulation of iron metabolism is posited to avert cognitive impairments, Aβ accumulation, and the hyperphosphorylation of tau protein, which are typically induced by chronic sleep deprivation in C57BL/6 mice (Yang et al., 2022).

In the future, these drugs may be further optimized to regulate IRPs more effectively, thus providing more therapeutic options for patients with neurodegenerative diseases (**Table 2**).

**Table 2:** The three main treatment strategies in the current study

Therapeutic	Targeted mechanism	Reference
Iron chelators	Regulate the activity of iron regulatory proteins and reduce iron accumulation	Devos et al., 2020
Iron responsive element inhibitor	Reduce ferritin and transferrin expression to attenuate excess iron accumulation	Rogers et al., 2011; Zhou and Tan, 2017
Antioxidant	Reduce oxidative stress, protect neurons	Teleanu et al., 2022
Ketogenic diet	Modulate key iron-related proteins	Tao et al., 2022; Yang et al., 2022

#### Iron metabolism-based disease markers

Given the central role of iron homeostasis imbalances in neurodegenerative diseases, iron metabolism-based biomarkers should be developed in the future for the early diagnosis and monitoring of disease progression. These markers may include the serum levels of ferritin, transferrin, or other iron metabolism-related proteins.

#### Application of multi-modality approaches

Multi-modality approaches refer to strategies that combine various research techniques and tools to gain a comprehensive understanding of complex biological systems and their interactions. Such approaches include imaging techniques, biochemical analyses, molecular biological analyses, and animal models, among other research methods (Salvi et al., 2024). When studying the relationship between iron homeostasis and neurodegenerative diseases, scientists can integrate multiple research technologies to more thoroughly explore the function of iron in the nervous system and its role in diseases, thereby promoting the development of new therapeutic strategies. Future research is expected to further elucidate the complex mechanisms between iron homeostasis and neurodegenerative diseases and ultimately provide patients with better diagnostic and treatment options.

## Conclusion

Iron plays an indispensable and crucial role in vital life activities, and there is an intricate connection between iron homeostasis and neurodegenerative diseases. Maintaining iron homeostasis is a key step in the preservation of normal neuronal functions. An imbalance in intracellular iron is directly related to the strength of a neuron’s antioxidant capacity, which in turn affects the survival status of neurons and their ability to resist oxidative-stress-related damage. IRPs, key regulatory factors of intracellular iron homeostasis, play crucial roles in the occurrence and development of neurodegenerative diseases. They can considerably affect intracellular iron levels by finely regulating their own expression and activity, which is crucial for maintaining the normal physiological functions of neurons. Therefore, by precisely regulating IRPs, it is possible to effectively intervene in neurodegenerative diseases. Studies of the changes in iron homeostasis that occur in neurodegenerative diseases have greatly broadened our understanding of the complex pathological mechanisms of these diseases. Disruptions to iron metabolism can lead to oxidative stress, inflammation, and cell death through processes such as ferroptosis. Research has not only revealed the close relationship between iron metabolism imbalance and neurodegenerative diseases but also provided potential targets for exploring new therapeutic strategies. Although considerable progress has been made in elucidating the mechanisms by which iron imbalance contributes to neurodegenerative diseases, several challenges remain. Future research should focus on developing more precise diagnostic tools to detect iron dysregulation in the early stages of the disease and designing targeted therapies that can effectively restore iron homeostasis while minimizing systemic side effects. Moreover, further explorations of the interactions between iron metabolism and other cellular processes, such as autophagy and protein aggregation, may reveal new therapeutic targets. Ultimately, any potential therapeutic targets should undergo rigorous clinical trials validation to ensure safety and efficacy. By continuously optimizing treatment plans, we have the potential to bring new hope to patients with neurodegenerative diseases, truly improve their quality of life, and contribute to the development of this field.

## Data Availability

*Not applicable*.
